# Combined training in addition to cortisol reduction can improve the mental health of girls with precocious puberty and obesity

**DOI:** 10.3389/fped.2023.1241744

**Published:** 2023-11-10

**Authors:** Ali Heidarianpour, Elnaz Shokri, Efat Sadeghian, Fatemeh Cheraghi, Zahra Razavi

**Affiliations:** ^1^ Faculty of Sports Sciences, Bu-Ali Sina University, Hamedan, Iran; ^2^ Department of Nursing, School of Nursing and Midwifery, Chronic Disease (Home Care) Research Center, Hamadan University of Medical Sciences, Hamadan, Iran; ^3^ Department of Pediatric Nursing, School of Nursing and Midwifery, Chronic Disease (Home Care) Research Center, Hamadan University of Medical Sciences, Hamadan, Iran; ^4^ Department of Pediatrics Endocrinology, School of Medicine, Besat Hospital, Hamadan University of Medical Sciences, Hamadan, Iran

**Keywords:** combined training, cortisol, mental health, obesity, central precocious puberty

## Abstract

**Background:**

Obesity and central precocious puberty (CPP) are associated with increased anxiety, depression, and anger in girls. The contribution of exercise as an efﬁcacious component in decreasing anxiety, depression, and anger has been increasingly recognized.

**Objectives:**

This study aims to evaluate the effects of combined training on cortisol, anxiety, depression, and anger in overweight and obese girls with CPP.

**Methods:**

The study involved 30 girls aged 7–9 years diagnosed with CPP (undergoing triptorelin treatment) and dealing with obesity. In addition, these girls scored higher than the cut-off line for anxiety, depression, and anger. The participants were divided into two groups, with 15 individuals in each group. The exercise group engaged in 60 min of combined aerobic and resistance training three times per week for a duration of 12 weeks. On the other hand, the control group did not receive any training. Throughout the study, the serum cortisol levels were measured in both groups. Anxiety, anger, and depression questionnaires were also completed at three different stages, namely, baseline, 12 weeks, and 16 weeks (after a 4-week period of detraining).

**Results:**

In the exercise group, there was a significant decrease (*P* < 0.05) in cortisol serum levels and anxiety, depression, and anger scores. These changes were observed consistently during detraining (*P* > 0.05). However, in the control group, only the depression score significantly decreased (*P* < 0.05).

**Conclusions:**

Based on the results, it can be concluded that combined training is a method to improve the mental health of CPP girls.

**Clinical Trial Registration:**

https://en.irct.ir/trial/61990, identifier IRCT20170411033378N10.

## 1. Introduction

In 2019, the World Health Organization (WHO) reported that approximately 38.2 million children below the age of 5 years were impacted by obesity. In 2016, over 340 million children and adolescents aged 5–19 years were affected by obesity (1). Obesity in children leads to various issues, including the early onset of puberty in girls (2). A retrospective, case–control study examining a large population of children over a 2-year period concluded that overweight and obesity among children, especially in girls, are related to increased odds of central precocious puberty (CPP), and these CPP-predisposing effects are found when overweight/obesity was prolonged for greater than 1 year for girls and 2 years for boys (3). Precocious puberty means the onset of puberty before the age of 8 in girls and 9 in boys (4). Precocious puberty has been linked to short stature, depression, aggression, social withdrawal, and moodiness (5). Also, early pubertal maturation is associated with anxiety symptomatology (6). The relationship between stress and depressive disorders has often been attributed to a dysregulation of the hypothalamic–pituitary–adrenal (HPA) axis (7). Moreover, the HPA axis regulates both the production and secretion of cortisol (8). Abdominal obesity is also associated with long-term elevated cortisol levels, followed by certain mental disorders (9). Therefore, precocious puberty and obesity should be prevented and treated.

The treatment for precocious puberty is gonadotropin-releasing hormone analog (GnRHa) (10). Training is highly effective for weight loss and preventing and treating obesity (11). Girls with a reduced body mass index–standard deviation score (BMI-SDS) experienced a delayed onset of puberty compared with overweight girls who did not have a reduction in BMI-SDS (12). Recent studies have shown that physical activity can positively impact physical and mental health (13). Physical activity promotes improvements in depressed mood (14), anxiety (15), and aggression (16). Physical activity is a long-term lifestyle behavior that should be introduced in some capacity to all able children, adolescents, and adults (17). Using exercise as a stress management technique has been shown to have preventative and treatment effects on stress (18). Researchers have recommended that an individual exercise for 15–30 min at least three times a week in programs of 10 weeks or longer to reduce depression and anxiety (19). In addition, a study revealed that exercise substantially impacted the reduction of cortisol levels (20).

While training offers numerous advantages, individuals may also encounter detraining, resulting in the potential loss of adaptations and performance gained through training (21). The authors’ previous research showed that aerobic training reduces cortisol levels, and detraining eliminates the benefits of training in girls with precocious puberty (22).

While it is widely accepted that training can enhance the quality of life, a lack of sufficient information regarding the impact of combined training (aerobic + resistance) and detraining on the mental wellbeing of girls experiencing precocious puberty remains. If the effects of training and detraining on these girls are comprehended, these children and their families can make informed decisions about whether or not to engage in training programs. Therefore, our study aimed to assess the impact of a combination of training and detraining on depression, aggression, anxiety, and cortisol levels in overweight and obese girls experiencing precocious puberty.

## 2. Materials and methods

A pediatric endocrinology and metabolism specialist from Hospital Beasat in Hamadan City referred 75 girls with precocious puberty to our department of pediatrics. The selected girls received an intramuscular injection of GnRH analog (triptorelin) at a dose of 80 mg/kg (maximum: 3.75 mg) every 28 days. Only those children who met the inclusion and exclusion criteria of the study were chosen. The hormonal criteria for the diagnosis of CPP include using luteinizing hormone (LH), which is the best biochemical parameter to diagnose CPP. An increase in levels of LH greater than 0.2 IU/L can be considered a pubertal value (23). A provocative test with GnRH agonist with precise cut-offs is challenging to establish. A peak stimulated LH of >∼8 mIU/ml after GnRH and >∼5 IU/L after GnRHa indicates CPP (24).

The inclusion criteria include being overweight or obese based on the tri-ponderal mass index (TMI), falling within the age range of 7–9 years, having been diagnosed with precocious puberty at least 1 year prior, receiving treatment with the triptorelin drug, taking 1 ml of medication every 28 days, and achieving scores above 50 on the Spence Children’s Anxiety Scale, above 14 on the Birleson Depression Self-Rating, and above 95 on the Children’s Inventory of Anger.

The exclusion criteria include having another illness, taking another medicine, having a special diet, being active in another sport, and using narcotics.

**Figure 1:**
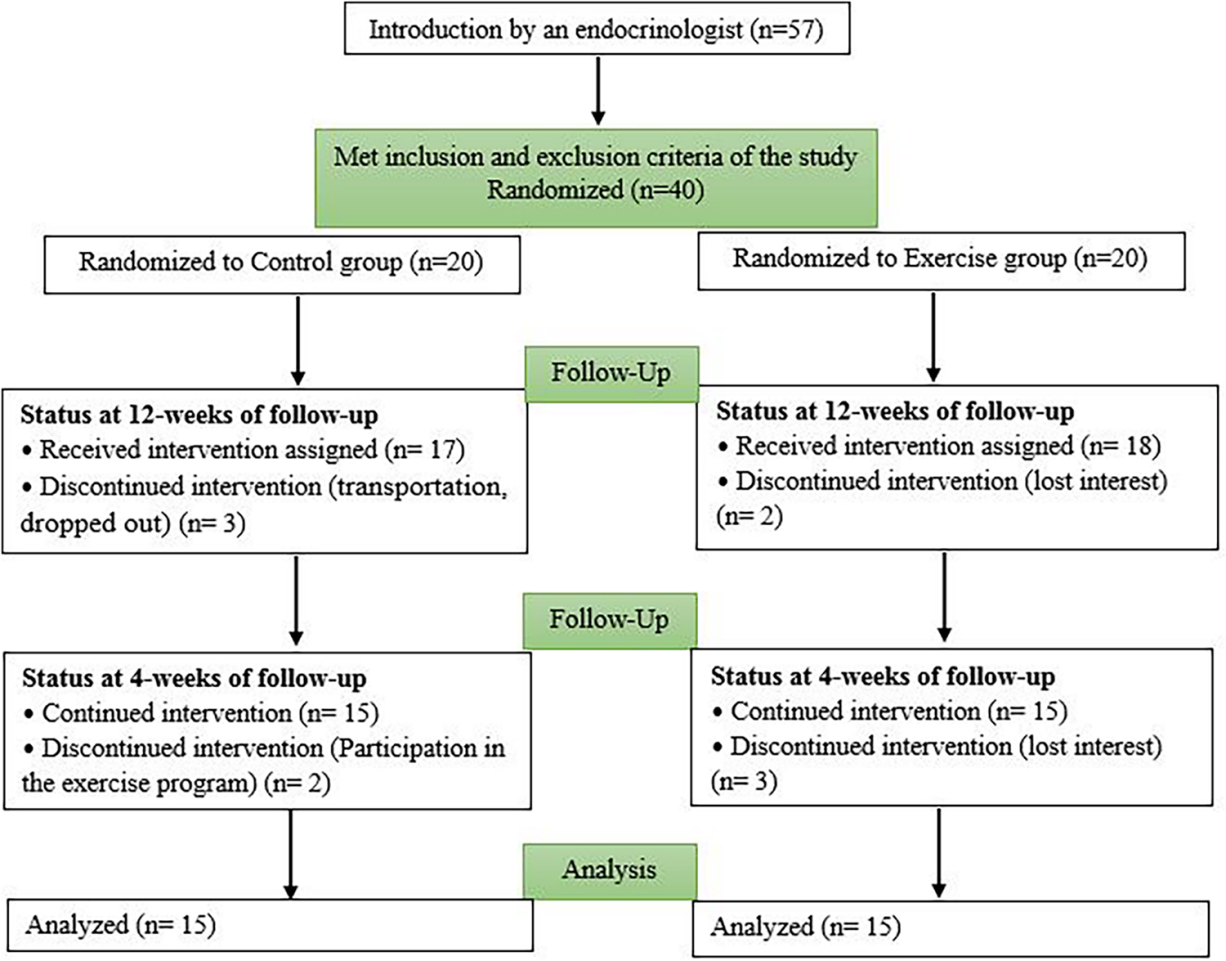
Follow the diagram of the study.

A total of 40 girls who met the inclusion and exclusion criteria were selected. After obtaining informed and written consent from their parents, they were randomly assigned to two separate groups. The exercise group (Ex) participated in a 12-week combined training program (*n* = 20), and the control group (Con) did not receive any exercise intervention (*n* = 20). Some were excluded from the study because of lack of interest, transportation, and participation in another sports program (Figure 1). Finally, 30 subjects were analyzed (Figure 1). The Research Ethics Committees of Hamadan University of Medical Sciences approved this research on 5 February 2022, with proprietary ID IR.UMSHA.REC.1400.883. The Iranian Registry of Clinical Trials also approved the study on 27 February 2022 IRCT20170411033378N10.

### 2.1. Measuring anthropometric indicators and blood samples

All children underwent anthropometric measurements conducted by two experienced physical education teachers. The height of the children was measured using a stadiometer, and their weight was measured using digital weighing scales. Waist circumference (WC, cm) and hip circumference (HC, cm) were measured using an elastic measuring tape, following the WHO recommendations (25). TMI was measured by body weight divided by height cubed. For children aged <16 years, the TMI thresholds to diagnose overweight status were 13.1 ≤ TMI ≤ 14.1 kg/m^3^, and the TMI thresholds to diagnose obese status were TMI ≥ 14.1 kg/m^3^ (26). Using a caliper, skinfold thickness was measured from the left side of the body to the nearest 0.1 mm in two places: (1) triceps, halfway between the acromion process and the olecranon process, and (2) subscapular, approximately 20 mm below the tip of the scapula, at an angle of 45° to the lateral side of the body (27). To increase the accuracy, the measurement was repeated three times. To measure muscle mass, the method of Poortmans et al. was used, which has been validated in children and adolescents (28). Peak oxygen uptake (VO_2_peak) was measured using the 6 min walk test (6MWT) according to the American Thoracic Society rules. The test was performed in a 30 m flat corridor, where contributors were advised to walk as fast as they could without running for 6 min, and they were also encouraged with standard motivational statements (29). This test has already been administered to and validated on children (30). To measure systolic blood pressure (SBP) and diastolic blood pressure (DBP), an automatic barometer was used. Immobile participants were seated during the measurement. To reduce error, the measurements were repeated twice. The watch fastened to the wrist measured the resting heart rate (RHR).

A laboratory specialist collected blood samples between 8:30 and 9:00 a.m. after a fasting period of 12 h, within 2 h of waking up. The samples were taken from the ulnar vein and placed in optocoupler coagulation tubes purchased from Italy. After the blood clotted, the clot was separated by centrifugation at a speed of 1,000–2,000×*g* for 10 min, resulting in the extraction of the serum. A laboratory kit was used to measure cortisol serum levels (BOSTER, Canada) with a 0.5 ng/ml sensitivity for the enzyme-linked immunosorbent assay (ELISA) method.

### 2.2. Measuring mental health variables

Children’s anxiety was assessed using the Spence Children’s Anxiety Scale child (SCAS-C) (31). This questionnaire appraises six domains of anxiety, namely, separation anxiety, social phobia, obsessive–compulsive disorder, panic, generalized anxiety, fears, and physical injury. This scale is easy for children and will be completed in 10 min. This questionnaire has 44 items, of which 38 reflect specific signs of anxiety and six relate to positive filler items to reduce negative response bias. Of the 38 anxiety items, six items reflect separation anxiety, six social phobias, six obsessive–compulsive problems, nine panic, six generalized anxiety, and five fears of physical injury. Items are haphazardly allocated within the questionnaire. Anxiety is rated on a 4-point scale from never (0) to always (3). *T*-scores and cut-offs for the SCAS-C are available on the SCAS website according to age and gender. Using the SCAS-C, for girls aged 8–11 years, a total cut-off score of ≥50 is suggested. There are no suggested cut-offs for children aged 7 years, so the threshold of ≥50 was used for girls of this age. The Persian version of SCAS has acceptable reliability and validity for Iranian children and adolescents (32).

Children’s depression was assessed using the Birleson Depression Self-Rating Scale for Children (DSRS-C). This scale is an 18-item inventory appropriate for children and adolescents aged between 8 and 14 years. Participants must choose one of three options, namely, “most of the time,” “sometimes,” or “never.” The cut-off point of this questionnaire for girls is 14 (33). The reliability and validity of the DSRS-C scale were considered satisfactory (34).

Children’s anger was evaluated using the Children’s Inventory of Anger (ChIA) (35). It has 39 items used to evaluate children aged 6–16 years. This tool measures four subscale scores, namely, frustration, physical aggression, peer relationships, and authority relations. The internal reliability for the total scale is reportedly high (α = 0.95; subscale coefficients ranged from 0.85 to 0.87), and the 1-week test–retest reliability was 0.75 (35).

### 2.3. Frequency of measurements

All anthropometric indices, blood samples, anxiety, depression, and anger were measured three times from all subjects. Initial measurements were performed 1 day prior to the initiation of the exercise program. The second stage of measurements in the patients in the experimental and control groups was started 48 h after the last exercise session to evaluate the effect of the exercise program on the mentioned indices. The third stage of measurements was performed following the 4-week detraining period. The decision to select a 4-week detraining period was based on the findings that a mere 2-week detraining period is insufficient to diminish the positive impacts of regular exercise fully. However, prolonged detraining may have detrimental consequences (36).

### 2.4. Intervention

The exercise program was conducted in a gym and supervised by two experienced physical education instructors. The first session was dedicated to measuring anthropometric indicators and familiarizing participants with exercise. The training protocol was performed for 12 weeks, with three sessions per week and 60 min of aerobic + resistance training in each session according to the Lambert protocol (37). Exercise sessions consisted of 30 min of aerobic exercise followed by 20 min of strengthening exercises and 10 min of stretching and cool-down. To promote physical activity in children of different age groups, it is advisable to incorporate unstructured play for young children and engaging activities for older children (38). The aerobic exercise section used fast walking, running, and ball games. Subjects played all the indicated exercises in all sessions at a heart rate corresponding to 55%–65% of individual maximal cardiorespiratory fitness (based on baseline VO_2_peak measures by 6MWT). In every training session, children were equipped with a heart rate monitor. If their heart rate dropped below 55% or went above 65% of their VO_2_peak, watch alarms would alert them. The aerobic period was followed by strengthening exercises of the arms, legs, and trunk (two to three series of 10–15 repetitions), with the resistance being provided by the body weight of the child and elastic bands (37). The Con did not receive any interventions, and the researchers asked them not to participate in any exercise activities. After the 3-month intervention, participants were asked not to participate in any exercise programs for 4 weeks.

### 2.5. Statistical analysis

During the intervention, we utilized the analysis of variance (ANOVA) with repeated measures test to identify significant changes in the difference between groups and within groups. To evaluate the relationship between the variables, we used the Pearson correlation coefficient. All analyses were performed using SPSS 20 software. The findings were declared as mean ± SD. Differences were assigned statistically at *P* < 0.05.

## 3. Result

### 3.1. Effect of the combined training and detraining on anthropometric and clinical parameters

Table 1 presents the individual physiological and anthropometric characteristics of the participants in the three stages (baseline, 12 weeks, and 16 weeks). The results confirm that 12 weeks of combined training decreased the weight, TMI, WC, subscapular skinfold thicknesses (SS), RHR, and SBP and increased the 6MWT, VO_2_peak, and muscle mass (*P* < 0.05). Although the detraining period led to increased weight, TMI, WC, and SS, their values did not reach the initial levels before training. 6MWT and VO_2_peak returned to their initial levels during the detraining period. Muscle mass and SBP did not change during the detraining period.

**Table 1:** Subject’s physiological and anthropometric characteristics in two groups (exercise and control) and three stages (baseline, 12 weeks, and 16 weeks).

Parameters	Group	Baseline	12 weeks	16 weeks	*F*
Age (year)	Ex	8.26 ± 0.37	8.51 ± 0.44	8.59 ± 0.75	0.33
	Con	8.1 ± 0.42	8.35 ± 0.59	8.43 ± 0.8	0.41
Height (cm)	Ex	135.2 ± 7.72	135.8 ± 7.83	136.2 ± 7.91	2.75
	Con	135.4 ± 8.24	135.9 ± 8.49	136.3 ± 8.88	2.66
Weight (kg)	Ex	41.21 ± 2.19	39.07 ± 2.38^a,c^	39.87 ± 2.26^a,b,c^	48.53
	Con	41.0 ± 2.55	41.75 ± 2.31	41.96 ± 2.6	0.67
TMI (kg/m^3^)	Ex	16.4 ± 2.45	14.63 ± 1.79^a,c^	15 ± 1.88^a,b,c^	89.76
	Con	16.19 ± 2.38	16.24 ± 2.4	16.31 ± 2.5	0.32
WC (cm)	Ex	69.44 ± 13.27	68.29 ± 12.96^a,c^	68.58 ± 13.27^a,b,c^	18.36
	Con	69.35 ± 11.84	69.47 ± 12.18	69.49 ± 12.59	1.14
WHR	Ex	0.77 ± 0.07	0.77 ± 0.08	0.77 ± 0.08	0.73
	Con	0.76 ± 0.06	0.76 ± 0.08	0.76 ± 0.09	1.55
TS (mm)	Ex	18.8 ± 6.39	16.25 ± 5.66	17.72 ± 6.24	2.79
	Con	18.42 ± 7.19	18.48 ± 5.75	18.5 ± 6.09	0.51
SS (mm)	Ex	16 ± 5.41	14.1 ± 4.65^a,c^	14.77 ± 5.05^a,b,c^	65.07
	Con	15.67 ± 5.33	15.84 ± 5.46	15.87 ± 5.55	0.49
Muscle mass (kg)	Ex	18.4 ± 4.21	19.7 ± 4.59^a,c^	19.3 ± 4.45^a,c^	33.85
	Con	18.8 ± 3.38	18.9 ± 4.01	18.9 ± 4.27	1.77
6MWT (m)	Ex	557 ± 52.29	674 ± 58.25^a,c^	600 ± 51.07^b^	91.12
	Con	563 ± 57.06	584 ± 50.11	597 ± 55.34	0.58
VO_2_peak (ml/kg/min)	Ex	37.14 ± 5.28	40.62 ± 6.48^a,c^	38.58 ± 5.44^b^	18.65
	Con	37.23 ± 6.17	38.38 ± 6.55	38.41 ± 6.42	0.34
RHR	Ex	101.26 ± 10.43	83.4 ± 9.85^a,c^	88.19 ± 10.43^a,b,c^	29.66
	Con	98.33 ± 11.57	99.71 ± 10.77	100 ± 10.64	1.11
SBP (mmHg)	Ex	118.49 ± 13.56	108.22 ± 12.39^a,c^	111.53 ± 13.77^a,c^	15.83
	Con	116.65 ± 14.27	118.2 ± 13.47	118.34 ± 13.19	0.26
DBP (mmHg)	Ex	80 ± 4.73	77.53 ± 4.35	78.83 ± 5.49	1.38
	Con	78.27 ± 5.15	80 ± 4.7	80 ± 5.21	0.54

Ex, exercise group, *n* = 15; Con, control group, *n* = 15; TMI, tri-ponderal mass index; WC, waist circumference; WHR, waist-to-hip ratio; TS, triceps skinfold thicknesses; SS, subscapular skinfold thicknesses; 6MWT, 6-minute walk test; VO_2_peak, peak oxygen uptake; RHR, resting heart rate; SBP, systolic blood pressure; DBP, diastolic blood pressure.

Data presented include the mean and standard deviation (SD).

*P* < 0.05.

^a^ Significantly different from baseline.

^b^ Significantly different from 12 weeks.

^c^ Significantly different from the control group.

### 3.2. Effect of the combined training and detraining on cortisol levels

**Figure 2:**
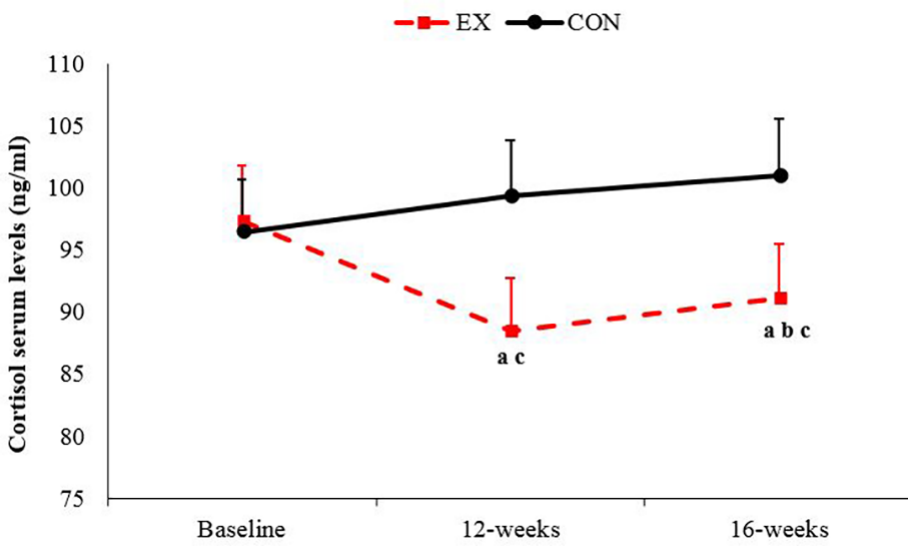
Changes in cortisol serum levels from the baseline to the end of 16 weeks of study in the exercise and control groups: (**a**) significantly different from baseline in the exercise group; (**b**) significantly different from 12 weeks; (**c**) significantly different between the exercise and control groups. *P* < 0.05. Ex, exercise; Con, control. Data are reported as mean ± standard deviation.

The results indicated that the training intervention decreased the cortisol concentrations, and those concentrations were maintained throughout the detraining period (*P* < 0.05). In the Con, no significant change was observed throughout the study (Figure 2).

### 3.3. Effect of the combined training and detraining on mental health indices

The results demonstrated that combined training led to a significant decrease in the overall anxiety score among children. However, in the Con, there were no significant differences in the total anxiety score, except for the panic and generalized anxiety subscales, which showed a notable decrease (Table 2).

**Table 2:** Mean and standard deviation of anxiety, anger, and depression scores in two groups (exercise and control) and three stages (baseline, 12 weeks, and 16 weeks).

Parameters	Subscale	Group	Baseline	12 weeks	16 weeks	*F*
Anxiety and its subscale scores	Separation anxiety	Ex	14.42 ± 1.4	10.35 ± 1.2^a,b^	10.71 ± 1^a,b^	14.08
		Con	13.77 ± 1.2	13 ± 2.6	12.45 ± 1.8	0.29
	Social phobia	Ex	10.83 ± 2.3	8.11 ± 1.5	8.42 ± 1.1	0.48
		Con	9.75 ± 0.7	9.63 ± 0.9	9.8 ± 1.4	1.62
	Obsessive–compulsive problems	Ex	7.87 ± 1.7	6.4 ± 0.6	6 ± 0.4	0.33
		Con	7.91 ± 0.5	6 ± 1.3	7.11 ± 0.6	2.48
	Panic	Ex	12.55 ± 1.3	10.46 ± 0.8^a^	11.35 ± 2.4	20.59
		Con	11.73 ± 0.8	10 ± 1.7	9.79 ± 1.9^a^	69.14
	Generalized anxiety	Ex	10.84 ± 0.7	7.31 ± 0.2^a,b^	7.11 ± 1.2^a,b^	80.48
		Con	11.62 ± 1.4	10.9 ± 1.8	9.18 ± 1.5^a^	65.38
	Fears of physical injury	Ex	9.22 ± 0.1	5.14 ± 0.4^a,b^	6 ± 0.3	11.43
		Con	11.8 ± 1.1	10.73 ± 1.8	9.25 ± 1.5	1.73
	Total score	Ex	65.73 ± 2.3	47.77 ± 2.6^a,b^	49.59 ± 2.9^a,b^	82.08
		Con	66.58 ± 3.4	60.26 ± 3.8	57.58 ± 4.1	3.55
Anger and its subscale scores	Frustration	Ex	29.44 ± 4.03	18.28 ± 3.65^a^	19.68 ± 2.93^a^	22.06
		Con	24.75 ± 4.24	21.14 ± 5.17	20.38 ± 3.66	0.31
	Physical aggression	Ex	27.36 ± 3.71	20.35 ± 3.75^a^	20.09 ± 2.29^a^	6.78
		Con	25.6 ± 5.44	20.77 ± 4.55^a^	20.47 ± 5.18^a^	19.54
	Peer relationships	Ex	27.19 ± 4.62	19.56 ± 2.71^a,b^	19.37 ± 4.19^a,b^	11.74
		Con	25.43 ± 3.55	23.18 ± 2.67	23.02 ± 3.88	0.39
	Authority relations	Ex	28.47 ± 3.33	21.54 ± 3.07^a,b^	22.39 ± 2.65^a^	69.16
		Con	25 ± 2.27	23.54 ± 5.15	22.68 ± 3.78	0.28
	Total score	Ex	112.46 ± 17.46	79.73 ± 16.66^a,b^	81.53 ± 19.47^a,b^	56.05
		Con	100.78 ± 16.09	88.63 ± 20.53	86.55 ± 20.27	1.19
Depression scores	Total depression scores	Ex	17.80 ± 3.22	12.45 ± 2.19^a^	11.27 ± 2.06^a^	89.28
		Con	16.73 ± 3.47	14.14 ± 3.65^a^	12.86 ± 3.27^a^	34.57

Ex, exercise group, *n* = 15; Con, control group, *n* = 15.

Data presented include the mean and standard deviation (SD).

*P* < 0.05.

^a^ Significantly different from baseline.

^b^ Significantly different between the exercise and control groups.

Furthermore, the results indicated a notable reduction in the depression scores among children who participated in a 12-week exercise program. Likewise, the Con, which received a 12-week treatment involving GnRH agonist, also witnessed a significant decrease in their depression scores (Table 2).

The statistical analysis of the data revealed a significant decrease in the total score of Nelson’s Anger Questionnaire with combined training. Conversely, no significant changes in anger scores were observed in the Con. However, the Con did experience a significant decrease in the physical aggression scores (Table 2).

### 3.4. Correlation analyses

**Figure 3:**
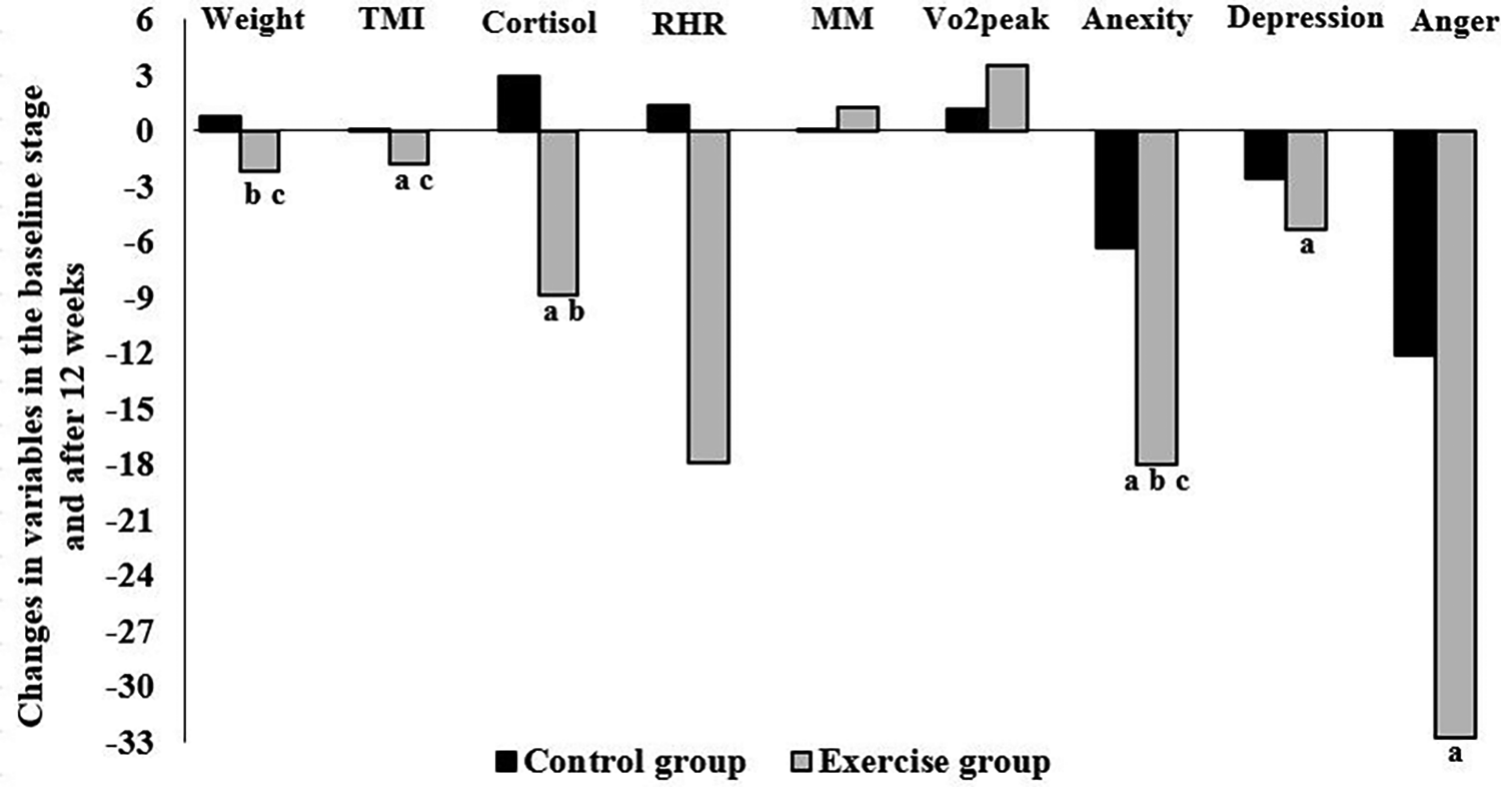
Correlation between the changes in variables in the baseline stage and after 12 weeks in the exercise and control groups: (**a**) significant correlation with weight, (**b**) significant correlation with TMI, and (**c**) significant correlation with cortisol. *P* < 0.05. TMI, tri-ponderal mass index; RHR, resting heart rate; MM, muscle mass; VO_2_peak, peak oxygen uptake.

Figure 3 shows the number of parameter changes between baseline and 12 weeks, as well as the significant Pearson correlation between them. A positive correlation existed between changes in weight and changes in TMI (*r* = 0.311), cortisol (*r* = 0.104), anxiety (*r* = 0.265), depression (*r* = 0.192), and anger (*r* = 0.209). Also, changes in serum cortisol levels were positively correlated with changes in TMI (*r* = 0.173) and anxiety (*r* = 0.164) (*P* < 0.05).

**Figure 4:**
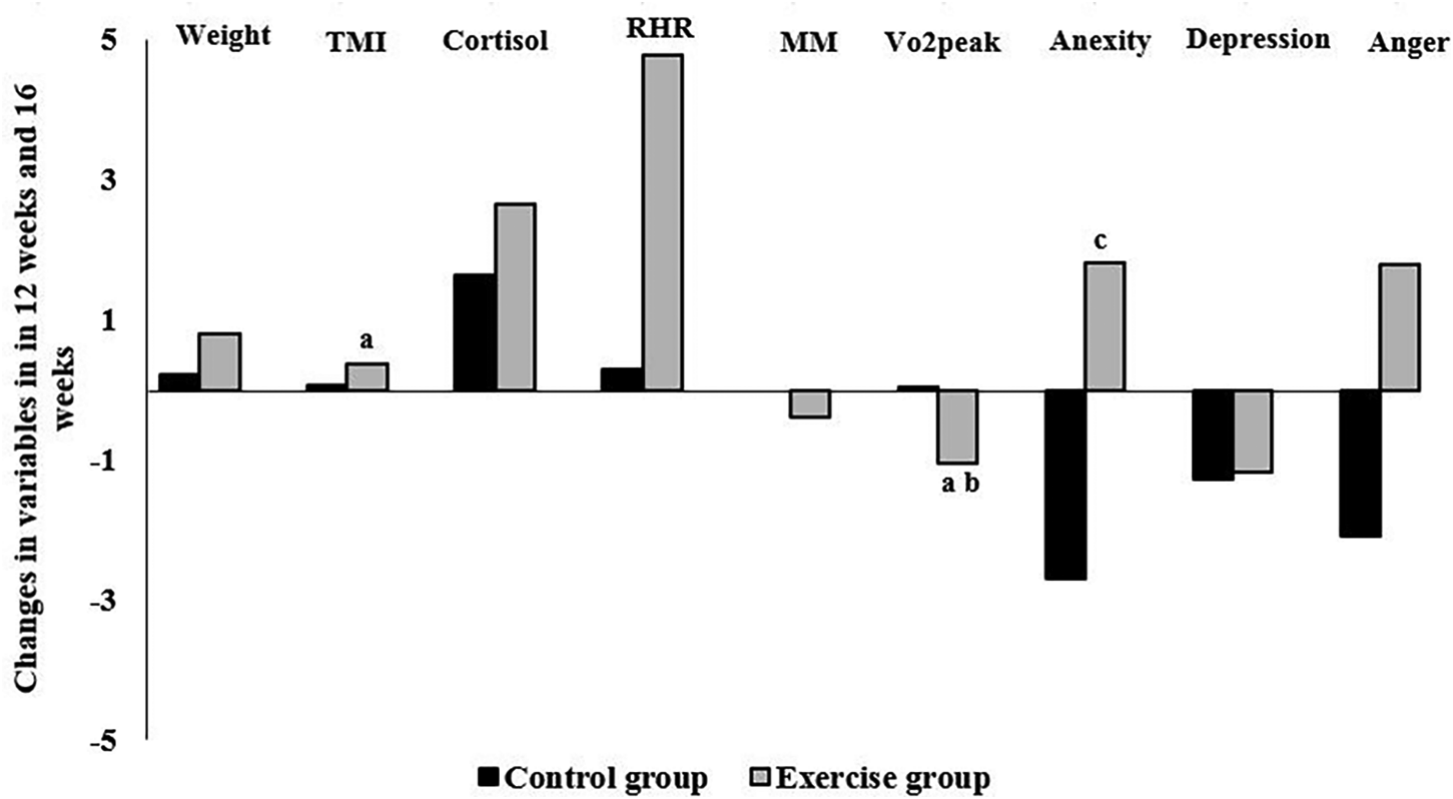
Correlation between the changes in variables in 12 weeks and 16 weeks in the exercise and control groups: (**a**) significant correlation with weight, (**b**) significant correlation with TMI, and (**c**) significant correlation with cortisol. *P* < 0.05. TMI, tri-ponderal mass index; RHR, resting heart rate; MM, muscle mass; VO_2_peak, peak oxygen uptake.

Figure 4 shows the number of parameter changes between 12 weeks and 16 weeks, as well as the significant Pearson correlation between them. As shown in Figure 4, there is a significant positive correlation between weight changes and TMI changes (*r* = 0.218), and there is a negative correlation between weight changes and VO_2_peak changes (*r* = −0.125). Also, there is a significant positive correlation between cortisol changes and anxiety changes (*r* = 0.14) (*P* < 0.05).

## 4. Discussion

As hypothesized, we found that 12 weeks of combined training led to a decrease in children’s anxiety, depression, and anger scores, as well as a decrease in serum cortisol levels. Meanwhile, in the Con, only depression decreased significantly. Furthermore, the positive effects of the combined training persisted during the 4-week detraining period. Potential mechanisms contributing to the reduction of the puberty onset age with obesity include the presence of aromatase in adipose tissue. Aromatase is an enzyme that can produce estrogens from adrenal androgen (39). Moreover, obesity is associated with insulin-induced reductions of sex hormone-binding globulin (SHBG) (40), which increases the bioavailability of sex steroids including estradiol. However, exercise causes weight loss through several mechanisms. Exercise can increase energy consumption and regulate energy balance, reduce body fat, improve body components, regulate insulin metabolism, and improve lipid metabolism. Obesity can also regulate the expression of genes and contend gene defects to some extent (41). The results of previous studies demonstrate that weight loss, reduced caloric intake, and catabolic state have a very powerful influence on the HPA axis and other endocrine systems (42). As observed in the Ex group during this study, the decrease in weight and TMI could potentially account for the reduction in cortisol levels within this group. Using TMI as a screening tool to identify obesity and overweight status in children and adolescents is worth contemplating (26). Therefore, we focused on TMI. Because of the limitation of BMI in distinguishing adipose mass from muscle, TMI has been proposed as a new indicator in assessing adiposity in children and adolescents better (43).

Another hypothesis to explain our results is to point out that at the level of the central nervous system, neuropeptides and corticosteroid receptors (glucocorticoid and mineralocorticoid receptors) in the brain and anterior pituitary play a major role in regulating circulating cortisol levels. Decreased pituitary sensitivity in endurance-trained athletes is one mechanism to reduce cortisol levels (20). Also, it has been shown in rats that restoring activity in the HPA axis with regular exercise resulted from adaptations in the hypothalamus and adrenal gland that promote a lower corticotropin-releasing hormone production and reduced adrenal sensitivity to adrenocorticotropic hormones, respectively (44).

Another point to discuss is that multi-facet exercises incorporating both aerobic and resistance training produce varied responses to cortisol production. The increase in VO_2_peak and muscle mass in the intervention group confirmed an expected effect of the aerobic and resistance training performed during the intervention period, respectively. It has already been reported that high cardiorespiratory fitness levels are associated with a lower diurnal cortisol output (45). In this research, VO_2_peak increased and had a negative relationship with cortisol, which could be a possible mechanism of cortisol reduction. On the other hand, cortisol is a catabolic hormone that stimulates degradation and inhibits the synthesis of muscle proteins (46). However, in our study, muscle mass increased due to combined training.

A recent meta-analysis has confirmed that physical activity provides significant benefits in reducing symptoms of depression, anxiety, and distress among different adult populations. These populations include the general public, individuals with mental health disorders, and those suffering from chronic diseases (47). The findings prove that exercise can have multiple beneficial effects on the physical and mental health of people with a wide range of mental disorders (48). The mechanisms behind the possible stress-buffering effects of exercise are still unclear. An early meta-analysis proved that aerobically fit subjects had a reduced psychosocial stress response (e.g., heart rate and blood pressure) compared with unfit individuals (15). It was previously argued that the benefits associated with training might be due to neurotransmitter, neuromodulator, and psychological mechanisms (49), oxidative stress, pro-inflammatory cytokines, and anti-inflammatory cytokines (7). In this regard, our previous research also confirmed that combined training and aerobic training increased adiponectin and decreased resistin and the signs of puberty (50, 51). Training may also increase body temperature, blood circulation in the brain, and impact on the HPA axis and physiological reactivity to stress (19).

Exercise has been found to have an antidepressant effect in both human and animal studies. This is achieved by boosting mitochondrial activity in brain neurons, promoting the release of monoamine neurotransmitters, increasing the levels of neurotropic factors, preventing the excessive production of inflammatory factors, and controlling the expression of microRNAs (14).

Serotonin has been linked to exercise and anger expression. In human adults, aggression has been shown to correlate negatively with the serotonergic activity of the central nervous system (52). In rats, exercise has been shown to buffer stress by altering serotonin and norepinephrine brain systems (53), so this may account for the reduced anger scores observed in the exercise condition. However, in the current study, serotonin was not evaluated.

A significant decrease in depression scales in Con is noted in our study, while anxiety and anger scores did not change during the 16-week treatment with GnRH agonist. In the same field, Yu et al. stated that a longer follow-up duration may lead to a stronger improvement in more patients with clinical psychological issues from a larger pool (54).

## 5. Conclusion

Overall, it can be concluded that overweight and obese children with CPP who participated in the training program experienced a decrease in cortisol, anxiety, depression, and anger compared with the Con. For overweight girls with CPP, engaging in physical activity alongside GnRH treatment not only improves physiological aspects like weight, heart rate, and cardiorespiratory fitness but also enhances psychological wellbeing. However, treatment with GnRH alone does not provide the same benefit levels. In addition, the effects of training on psychological and physiological characteristics were not completely eliminated during the 4-week detraining period, except for VO_2_peak. Exercise interventions should be integrated into the regular care of girls with CPP to promote physical and mental health.

## 6. Limitations

Some limitations of our study merit consideration. The sample size was small. The impact of diet, chemicals, and industry was not controlled, and the changes in the dietary or physical habits of the participants were not monitored in this study. Another limitation of the study was the inadequacy of the measurement of cortisol alone as a stress marker.

## Data Availability

The raw data supporting the conclusions of this article will be made available by the authors, without undue reservation.
